# Surgical Treatment of Atrial Fibrillation: Cutting Through the Edges

**DOI:** 10.21470/1678-9741-2019-0057

**Published:** 2020

**Authors:** Amer Harky, Christiana Bithas, Jeffrey Shi Kai Chan, Mostafa Snosi, Dimitrios Pousios, Andrew D Muir

**Affiliations:** 1 Department of Cardiothoracic Surgery, Liverpool Heart and Chest Hospital, Liverpool, UK.; 2 School of Medicine, University of Liverpool, Liverpool, UK.; 3 Faculty of Medicine, The Chinese University of Hong Kong, Shatin, New Territories, Hong Kong.; 4 Division of Cardiology, Department of Medicine and Therapeutics, Prince of Wales Hospital, Shatin, New Territories, Hong Kong.

**Keywords:** Atrial Fibrillation, Heart Rate, Heart Surgery, Forecasting, Catheter Ablation

## Abstract

Medical management of atrial fibrillation can be complex, challenging and requiring time to prove its effectiveness; furthermore, the response can be refractory and inconsistent if the underlying pathology is not permanently addressed. Surgical ablation has become a key intervention, and since its first intervention in 1987 (the Cox-maze procedure), the technique has evolved from a conventional open method to a minimally invasive technique whilst retaining excellent outcomes. Furthermore, recent advances in the use of a hybrid approach have been established as satisfactory approach in managing atrial fibrillation with satisfactory outcomes.

This literature review focuses on the evidence behind the surgical success in managing atrial fibrillation throughout the past, present and the future of these surgical interventions.

**Table:** 

Abbreviations, acronyms & symbols	
AF	= Atrial fibrillation
CPB	= Cardiopulmonary bypass
GP	= Ganglionic plexus
LAA	= Left atrial appendage
LAD	= Left atrial diameters
PVI	= Pulmonary vein isolation
PV	= Pulmonary vein
RF	= Radiofrequency
SR	= Sinus rhythm

## INTRODUCTION

AF is defined as a supraventricular arrhythmia which is characterised by uncoordinated atrial activation with consequent deterioration of atrial mechanical function. It can be further subdivided into differing types, as summarized in Table 1.

**Table 1:** Subdivisions of atrial fibrillation.

**First diagnosed AF**	Patients first presenting with AF, irrespective of the duration of arrhythmia or the presence and severity of AF-related symptoms
Paroxysmal AF	Self-terminating AF (usually within a 48-hour period). AF paroxysms may persist for up to 7 days. After 48 hours, patients' likelihood of spontaneous conversion is low and anticoagulation should be considered
Persistent AF	An episode of AF lasting >7 days or requiring termination by cardioversion, either with drugs or by direct current cardioversion
Long-standing persistent AF	AF present for a period of one year or more, when it is decided to adopt a rhythm-control strategy
Permanent AF	AF is accepted by the patient and by the physician diagnostically. Here, rhythm-control interventions are by definition not pursued in patients with permanent AF. If a rhythm-control strategy is adopted (such as considering AF surgery), the arrhythmia is redesignated as 'long-standing persistent AF'

The risks associated with having atrial fibrillation (AF) are wide and vary from causing immediate haemodynamic compromise to thromboembolic complications which can be incapacitating or even lethal and catastrophic^[1]^.

Atrial fibrillation is prevalent in 1-2% of the general population, and this figure increases with age and presence of concurrent heart disease^[1]^. It is estimated that as many as 5% of patients undergoing cardiac surgery have a coexisting diagnosis of preoperative AF, which in turn has a direct influence on their postoperative morbidity and mortality rates^[2]^.

Antiarrhythmic drugs, catheter-based ablation and surgery have all been proposed as means to manage AF, however they all vary in efficacy and accessibility^[2]^. Several randomized controlled trials and, lately, three systematic review and meta-analyses studied the effect of catheter-based ablation *versus* medical therapy in patients with paroxysmal or persistent symptomatic AF^[3-12]^. The overall conclusion from all these studies is the proven superiority of catheter-based ablation over medical therapy in terms of maintaining long-term sinus rhythm and better quality of life.

Surgical treatment for AF was effectively carried out for the first time in 1987 through the Cox-maze I procedure, which is characterised by its ‘cut and sew’ approach. This technique currently claims a success rate in sinus rhythm of 97-99%^[13]^. It was the first procedure to address all three detrimental consequences of AF: restoring the synchronicity of heart rhythm, promoting a regular ventricular response and decreasing the risk of thromboembolism and stroke^[2]^. However, the disadvantage of such technique was its inability to produce appropriate sinus function and postoperative left atrial dysfunction^[14]^.

Since then, alternative surgical approaches have been developed in an attempt to simplify the procedure and overcome technical challenges^[2,14]^. The initial ‘cut and sew’ approach of the Cox-maze I procedure to produce scar has been replaced by ablation techniques using other modalities such as radiofrequency (RF) ablation, cryotherapy, microwave, laser energy, high energy focused ultrasound, ganglionic plexus ablation, left atrial appendage (LAA) exclusion, N-contact ablation, and hybrid procedures^[2]^.

These techniques have reformed the surgical approach to AF management, which, in turn, has resulted in an increase in the number of patients undergoing AF correction procedures. Prior to the year 2000, less than 1% of patients submitted to cardiac surgery underwent Cox-maze procedure; however, and due to advancement in AF surgery, currently 40% of patients with known AF and undergoing cardiac surgery gets the concomitant ablation procedure^[15]^. Although these new ablation techniques have been shown to be safe and effective, care should be taken to select the most appropriate means of surgically managing AF. Further studies are still required to determine the long-term effectiveness of these new techniques, as some patients still develop recurrence of their AF postoperatively^[16]^.

At present, hybrid procedures seem to be the best solution at combining the advantages of both catheter and surgical ablation, such as confirming conduction block, the ability to close identified gaps that might lead to long-term recurrence and mitigating potential surgical injury to structures that are not easily reached. This is not applicable to all patients, however^[14]^.

### Indications for Surgical Ablations

The main indication for surgical intervention is symptomatic AF for all of its subtypes, ranging from persistent to permanent^[1]^. Surgery is often recommended for patients before the start of anticoagulation^[1]^. However, it is not recommended that such patients to have surgical intervention simply to avoid anticoagulation^[1]^. Other evidence-supported indications for surgery includes: increased quality of life, decreased stroke risk, decreased heart failure risk and improved survival^[1]^.

Current guidelines are less clear for patients considering stand-alone AF surgery^[1]^. They recommend that surgery should be offered only to symptomatic patients who are refractory or intolerant to at least one Class 1 or Class 3 antiarrhythmic drug^[1]^. Other indications of surgical interventions include failed catheter ablation and patient preference^[1]^.

The newest approach of hybrid thoracoscopic atrial fibrillation ablation is generally reserved for patients with paroxysmal AF refractory to medical therapy whose catheter ablation has failed, or for symptomatic patients with persistent or long-standing persistent AF^[17]^. However, it is contraindicated in patients with previous thoracic surgery, persisting AF for more than 10 years, a left atrial diameter greater than 6.5 cm, and a severely reduced left ventricular ejection fraction (<25%)^[17]^.

Following successful surgical or catheter-based intervention, anticoagulation therapy may be discontinued at 3 months provided that the patient is in sustained sinus rhythm^[1]^. However, this is only possible if the patient is deemed to be at low-risk for stroke^[1]^.

The 2012 HRS/EHRA/ESC guidelines outline a comprehensive overview of the indications for surgical ablation of AF; those are summarized in Table 2^[1]^. These recommendations are divided into two groups: patients undergoing concomitant surgical ablation together with other cardiac surgery, and patients undergoing stand-alone surgical ablation.

**Table 2:** Indications for surgical ablation together with other cardiac surgery.

**Symptomatic AF refractory or intolerant to at least one Class 1 or Class 3 antiarrhythmic drug**
Paroxysmal: surgical ablation is reasonable for patients undergoing surgery for other indications
Persistent: surgical ablation is reasonable for patients undergoing surgery for other indications
Long-standing persistent: surgical ablation is reasonable for patients undergoing surgery for other indications
**Symptomatic AF prior to initiation of drug therapy with a Class 1 or Class 3 antiarrhythmic drug**
Paroxysmal: surgical ablation is reasonable for patients undergoing surgery for other indications
Persistent: surgical ablation is reasonable for patients undergoing surgery for other indications
Long-standing persistent: surgical ablation may be considered for patients undergoing surgery for other indications
Indications for stand-alone surgical ablation of AF
Symptomatic AF refractory or intolerant to at least one Class 1 or Class 3 antiarrhythmic drug
Paroxysmal: stand-alone surgical ablation may be considered for patients who have not failed catheter ablation but prefer a surgical approach
Paroxysmal: stand-alone surgical ablation may be considered for patients who have failed one or more attempts at catheter ablation
Persistent: stand-alone surgical ablation may be considered for patients who have not failed catheter ablation but prefer a surgical approach
Persistent: stand-alone surgical ablation may be considered for patients who have failed one or more attempts at catheter ablation
Long-standing persistent: stand-alone surgical ablation may be considered for patients who have not failed catheter ablation but prefer a surgical approach
Long-standing persistent: stand-alone surgical ablation may be considered for patients who have failed one or more attempts at catheter ablation

AF=atrial fibrillation

### Factors Affecting Outcomes of Surgical Ablation

AF is induced by focal areas of enhanced autonomy in the atria mostly in and around the pulmonary veins, and less frequently around the superior vena cava and coronary sinus^[2]^. It is maintained by micro- and macro-re-entry circuits and also by tissue resonance of the fibrillar myocardium throughout the atria^[18]^, which becomes more persistent the longer the duration of AF^[2]^.

Ineffective atrial contraction not only reduces cardiac output by up to 30%, but also leads to blood stasis predisposing to thrombus formation, particularly in the LAA. As a result, treating paroxysmal AF should help in stopping the induction pathways, whereas treating permanent AF must address maintenance pathways. Despite this, factors such as surgical approach, method and patient profile all affect surgical outcomes.

### The Evolution of Cox-Maze Procedures

In 1987, the Cox-maze procedure was firstly performed in an attempt to eliminate atrial fibrillation through the use of incisional scars to block atrial macro-re-entry circuits that contribute with AF maintenance^[2]^.

This involved an extensive series of incisions that penetrated the walls of both atria and down into the septum, performed through a median sternotomy and requiring cardiopulmonary bypass (CPB)^[2]^. The Cox-maze procedure was designed to address all the adverse sequelae of AF and thus restored synchronicity, a regular ventricular response and reduced the risk of stroke and thromboembolism^[19]^.

Initially, in 1985 Cox proposed the initial atrial transection procedure and, despite its success in animals, it was unsuccessful during a human trial^[20]^. This technique, however, resulted in the subsequent development of the Cox-maze procedure^[2]^.

Since its development, adjustments have allowed the finalisation of the Cox-maze II procedure, which is currently widespread known as the “gold standard” surgical approach for AF^[21]^. Table 3 outlines a summary of the Cox-maze procedures and its modifications from previous iterations.

**Table 3:** Summary of the Cox-maze procedures and its modifications from previous iteration.

Procedure	Modification from previous iteration	Limitations of the procedure
Cox-maze I	NA	Inability to produce appropriate sinus tachycardia
(cut and sew)		Postoperative left atrial dysfunction
Cox-maze II	Left atrial: transverse atriotomy across the dome of the left atrium moved posteriorly	Prolonged intra-atrial conduction
(cut and sew)	Right atrial: elimination of SVC to right atrial lesion	Must completely transect SVC to gain left atrial exposure
Cox-maze III	Right atrial: placement of septal incision posterior to the orifice of the SVC	Prolonged CPB times and technical difficulty
(cut and sew)		
Cox-maze IV	Combination of bipolar RF ablation and cryoablation	Continued need for CPB
(bipolar RF ablation and cryoablation)	Left atrial: box lesion around posterior left atrium	

CPB=cardiopulmonar bypass; RF=radiofrequency; SVC=superior vena cava

In a study by Prasad et al.^[22]^, of 198 patients that underwent Cox-maze procedure, 97% of them were symptom-free after the procedure, and thereafter, several other studies have replicated those outcomes^[23,24]^. As means of increasing the effectiveness of the procedure, operative times have decreased over time, without altering the benefits of the traditional Cox-maze III procedure^[2]^.

This procedure is performed either through right mini-thoracotomy or a median sternotomy with requirement of CPB^[2]^. The right and left pulmonary veins (PVs) are grossly dissected to prepare for isolation. Patients may also be cardioverted with amiodarone allowing for determination of pacing thresholds on both sets of PVs before initiating ablation. The bipolar ablations are then carried out on a cuff of atrial tissue surrounding the right and left PVs individually. Once separated, exit block is confirmed with pacing from all the PVs. The pulmonary veins are an important source of ectopic beats, initiating frequent paroxysms of atrial fibrillation. These foci respond to treatment with radiofrequency ablation^[25]^.

The right atrial lesions are performed, while the heart is beating, through a single vertical atriotomy and a small purse-string suture at the base of the right atrial appendage. A unipolar source of energy, such as radiofrequency, is then utilised to finalise the ablation lines at the level of the tricuspid valve. After completion of the right side, the left-sided lesions are carried out through a standard left atriotomy with the heart arrested. The atriotomy is extended inferiorly around the right inferior PV and superiorly onto the left atrium^[2]^. A lesion is then performed with a bipolar RF device, connecting the left atrium incision at the bottom to the ablation line that encloses the left PVs^[2]^. An extra ablation is then performed from the superior aspect of the left atriotomy, across the dome of the left atrium and into the left superior PV^[2]^. A bipolar RF lesion is then carried up to extend to the mitral valve annulus. This lesion is performed from the bottom aspect of the left atrial incision across the posterior left atrium, AV groove, and the coronary sinus. The ablation is performed in the space between the circumflex and right coronary artery circulation to avoid compromising the coronary arteries.

To finalise the Cox-maze, a unipolar energy source is used to join the final ablation line to the mitral valve annulus. The LAA is amputated to decrease the risk of subsequent possible thromboembolism. A terminal ablation is then carried out through this amputated LAA and into one of the PVs. The LAA is then oversewn.

### Other Methods of Ablation

Development of technology and surgical techniques has led to the use of techniques that imitate the lesions of the Cox-maze III procedure without the need to penetrate through the full-thickness of the atrial walls. Those techniques are summarized below.

#### 1. Epicardial Radiofrequency Ablation

Radiofrequency ablation allows the creation of lesions using thermal energy to injure the targeted tissues^[2]^. As the radiation moves through the tissue, resistive heating takes place within a narrow edge of tissue in direct contact with the electrode. Passive conduction persists on this surface, forming the lesion in the deeper tissue. The RF ablation devices can be unipolar or bipolar. With unipolar catheters, the energy is distributed between the tip of the electrode and the indifferent electrode, usually the grounding pad applied to the patient. With bipolar means, an alternating current is creating, which leads to a more focused ablation. The size of the resulting lesion depends on the contact area of the tissue with the electrode, the temperature of the interface, the power and the duration. However, char formation may present as a challenge in achieving the desired deep tissue penetration. Irrigated catheters have been developed consequentially, as a means to overcome this^[25,26]^. RF ablation is well-known for its safety profile^[2]^. Complications associated with unipolar RF devices include coronary artery injury, cerebrovascular accidents and oesophageal perforation resulting in an atrio-oesophageal fistula^[27,28]^. Bipolar RF devices have removed the resulting collateral damage associated with unipolar devices and no clinical complication are yet to be reported in the literature^[2]^. An associated limitation is the requisite for the tissue to be clamped in the jaws of the device. This has restricted the potential lesion set, especially on the beating heart, and requires the use of adjunctive unipolar technology to create a compound lesion set.

##### A. Unipolar Radiofrequency Ablation

Current recommendations suggest that in patients undergoing cardiac surgery, concomitant unipolar RF ablation to treat AF is effective at restoring sinus rhythm (SR)^[1]^. The procedure is deemed safe in terms of not producing any additional risks^[1]^.

Unsuccessful ablations have been associated with patients with severe heart failure and left atrial diameters (LAD) exceeding 60 mm^[1]^. Furthermore, studies have shown that independent predictors of AF recurrence are LAD and age^[29-31]^.

In several studies, patients with differing types of AF have been shown to respond differently to unipolar RF ablation. Patients with paroxysmal or persistent AF had a higher rate of reverting to SR and sustaining it than those patients with permanent AF^[32]^. This, in turn, was associated with a decreased level of physical pain and improved health.

In terms of surgical approach, a study by Khargi et al. has suggested that there is a lack of correlation between the type of surgery performed and success rates^[33]^. In a further study by Maltais et al.^[34]^, they reported that the addition of unipolar RF ablation to open-heart surgery has not shown to cause an increase in mortality rates compared with undertaking the procedures alone, with SR being present in 71% of the 293 patients (71% for mitral surgery and 79% for coronary artery bypass grafting/aortic surgery, *P*=0.26).

Finally, Zangrillo et al.^[35]^ reported that unipolar radiofrequency ablation did not significantly increase cardiac troponin in comparison with mitral surgery alone (*P*=0.7)^[35]^.

Therefore, the use of unipolar radiofrequency ablation yields encouraging results, suggesting it is a favourable procedure to undertake in patients undergoing concomitant cardiac surgery for their AF ablation.

##### B. Bipolar Radiofrequency Ablation

Bipolar radiofrequency ablation has been shown to have a higher success rate in restoring SR in patients undergoing concomitant cardiac surgery, compared to patients receiving no ablation for a period of 1 year (75% *vs*. 30%, *P*=0.019)^[35]^. However, there is no current evidence suggesting that unipolar or bipolar methods are superior to each other^[1]^.

In a study by Chiappini et al.^[36]^, the reported survival rate was 97.1%, with 76% of patients being free from AF at a period of 13.8 months. However, the study by Srivastava et al.^[37]^ has shown that there is no statistical difference between biatrial maze and pulmonary vein isolation when considering the SR conversion rate. Bipolar radiofrequency procedures were also shown to have an extra cross-clamp time of 5 to 7 minutes^[37]^. Other studies have reported an extra cross-clamp time of 12 to 14 minutes^[38,39]^.

Another reported difficulty associated with using only bipolar ablation is the difficulty in guaranteeing a confluent ablation line between the left PVs, the mitral valve annulus and the tricuspid valve annulus without the risk of coronary artery involvement.

#### 2. Cryoablation

Cryoablation has been available for decades^[2]^. At present, there are two sources of cryothermal energy: argon and nitrous oxide. Nitrous oxide cryoablation has been extensively used on clinical base and has an unremarkable safety profile^[2]^. Cryoablation causes tissue injury through a process of freezing and rewarming. This microvascular damage leads to local tissue ischaemia. The size and depth at which cryoablation lesions are made depend on probe and tissue temperance, probe size, duration and number of ablations, and the particular liquid used as the cooling agent^[2]^. It has the distinct advantage of preserving the collagen structure, therefore preserving the fibrous skeleton of the heart, which aids in ensuring safety when being used around valvular tissue^[2]^. The potential disadvantages surrounding cryoablation include the length of time required to create a lesion (1-3 minutes), the challenge associated with creating lesions on the beating heart due to the circulating blood volume, and the risk of coagulation during epicardial ablation if frozen, which may instigate the onset of thromboembolism^[2]^.

Current recommendations suggest the use of cryoablation as an acceptable intervention for the treatment of AF with acceptable conversion rates of SR between 60 and 82% in one year^[1]^. It is noted to be more successful in patients with paroxysmal AF than those with permanent AF^[1]^. In a randomised multicentre trial by Budera et al.^[40]^, patients undergoing coronary artery bypass and/or valve surgery with AF were assigned to left atrial surgical ablation with an argon-based cryoprobe (group A) or no treatment for AF (group B); the right and left PVs were isolated separately, and then a connecting lesion, a mitral annulus lesion and a lesion to the LAA were performed and the appendage was removed. The SR rate was reported as 35.5% for the untreated group and 60.2% for the treated group (*P*=0.002). Stroke occurred in 2.7% (A) *versus* 4.3% (B) patients (*P*=0.319). No difference (A *vs*. B) in SR was found among patients with paroxysmal (61.9 *vs*. 58.3%) or persistent (72 *vs*. 50%) AF, but ablation significantly increased SR prevalence in patients with long-standing persistent AF (53.2 *vs*. 13.9%, *P*<0.001). No clinical benefits were shown in 1 year overall.

Another randomised controlled trial by Blomström-Lundqvist et al.^[41]^ showed that the use of cryoablation during mitral valve surgery had a higher complication rate than those that had mitral surgery alone. However, no significant impact was demonstrated regarding mortality or morbidity. The in-hospital complication rate was 11.4% in the mitral valve surgery group and 26.5% in the cryoablation group (*P*=0.110).

In a further study by Kim et al.^[42]^, the authors supported the recommendation that cryoablation may not be superior than other methods, such as microwave ablation, due to increased aortic cross-clamp time (*P*=0.005), and no differences in 3- or 5- years survival rates between patients having microwave ablation or cryoablation. The unadjusted 5-yr AF-free rate was 61.3±1.2% in the microwave ablation group and 79.9±3.2% in the cryoablation group (*P*=0.089).

Despite a high complication rate being reported with this technique, there was no overall change in long-term outcomes in terms of morbidity and mortality rates associated with cryoablation; therefore, the use of such technique remains debatable and at the discretion of the surgeon.

#### 3. Microwave Ablation

Microwave ablation involves the use of electromagnetic waves to generate heat by friction^[43]^. The subsequent release of heat creates lesions at predictable depth. The probe does not need to be in permanent contact with the tissue, proving favourable use, particularly in situations where achieving a complete dry field proves challenging intraoperatively^[43]^. The probe delivers energy which heats tissue to a depth of 6 mm without the risk of endocardial surface charring or coagulation^[43]^. Current recommendations have suggested that microwave ablation is less effective than other methods for the treatment of AF during concomitant cardiac surgery^[1]^.

Due to only one randomised study showing that outcomes for microwave ablation are inferior to RF ablation^[44]^, as well as limited other evidence, there are no devices currently on the market offering microwave ablation. This serves as a limitation to both understanding patient outcome and its effectiveness^[1]^.

In this randomised control trial, which sought to determine the effectiveness of microwave ablation, patients were treated with antiarrhythmic medication or were cardioverted during follow-up, interfering with the results, thus making it difficult to conclude whether microwave ablation had been effective^[44]^.

A study of 27 patients by Maessen et al.^[45]^ concluded that 87% of patients were in SR at a mean period of 6.4 months postoperatively. This supports microwave ablation not differing from radiofrequency ablation in terms of outcome.

Another study has demonstrated no difference in outcome with regard to freedom from AF with microwave ablation, with 80% of patients in the study being free from AF at 3 years and 61% being AF-free at 5 years^[42]^.

In a further study by Lin et al.^[46]^, they stated that the microwave antenna during the procedure had to be repositioned two or three times to finish the circular lesion around the endocardial pulmonary veins. The authors suggested that this uncertainty in transmurality and potentially the uninterruptedness of the lesion result in the inferior success rates associated with microwave in relation to RF ablation. This prospective trial concluded that RF was superior to microwave ablation, with more patients remaining in SR after RF ablation.

#### 4. Laser Energy Ablation

Laser energy is an efficient means of focusing energy to achieve tissue ablation using different wavelengths^[47]^. Laser energy allows the creation of focused, thin, well-demarcated lesions due to its reliance on conductive heat, allowing less energy to be dissipated^[47]^. As a result, it minimises collateral tissue damage. Due to its transparency when creating lesions, care should be taken to ensure that lesions are created continuously. A limitation associated with this technique is the increased risk of atrial thrombus formation^[47]^.

#### 5. High-Energy Focused Ultrasound

High-energy focused ultrasound permits an ablation device to be placed on the outside of the heart when delivering energy. This allows epicardial fat and myocardium to be ablated^[48]^. It does not damage the coronary arteries and as such it can be used to create a lesion across the left atrial isthmus from the epicardium without compromising the circumflex coronary artery. It enables contiguous transmural lines to be created and is compatible with minimally invasive techniques^[48]^. High-intensity focused ultrasound has proved to be ineffective in comparison to other devices and current recommendations do not support its use, as significant safety concerns have been reported^[1]^.

A high rate of failure has been reported by McCarthy et al.^[49]^ in their study of 408 patients who had 5 types of ablation procedures. It was found that only 43% of patients who underwent high-intensity focused ultrasound ablation were free from AF, compared to 90% with the maze procedure.

Complications reported in the literature associated with high-intensity focused ultrasound include late tamponade, postoperative haemorrhage requiring sternotomy, pericardial effusion, phrenic nerve palsies, injury to the oesophagus and atrio-oesophageal fistula^[50-52]^.

#### 6. Left Atrial Appendage Exclusion

LAA closure is performed either as a concomitant procedure during open-heart surgery or as a stand-alone surgical procedure as part of minimally invasive (minithoracotomy or thoracoscopy) arrhythmia surgery. LAA exclusion offers the possibility of decreasing the risk of thromboembolism to a level comparable with permanent anticoagulation. It also enables atrial booster function to be preserved^[53]^. Left atrial appendage (LAA) exclusion has been shown to have an increased risk due to poor surgical technique, leading to ineffective appendage exclusion^[1]^. As a result, it is recommended that, if contemplated, specialised devices fit for purpose should be used over approaching a cut-and-sew or stapling technique. In a study by Kanderian et al.^[54]^, LAA occlusion with excision was found to be more effective (73%) relative to suture (23%) and stapler exclusion (0%).

Furthermore, overall, LAA exclusion has been shown to have no proven benefit in taking into account outcomes such as stroke reduction or mortality benefit^[1]^. García-Fernandez et al., in their study of 205 patients that underwent LAA ligation procedure for AF, showed that 9.2% of patients had an ischaemic stroke^[54]^. The study found, however, that the absence of ligation of the LAA was an independent predictor of the occurrence of an embolic event following mitral valve surgery with OR of 6.7. The OR increased to 11.9 if the absence of effective ligation was incorporated into the model. Furthermore, in another study by Katz et al.^[55]^, a patient experienced a stroke one month after surgery. The study suggested that due to incomplete ligation that occurs during surgery, residual communication between the incompletely ligated appendage and the left atrial body may result in a milieu of stagnant blood flow within the appendage and is a mechanism of embolic and ischaemic events. It is important to note that thromboembolic events have also been associated with concomitant LAA exclusion in patients undergoing a mechanical mitral valve replacement, with 65% of a total of 72 patients experiencing one after having the LAA ligated^[56]^. The study by Bando et al.^[56]^ concluded that closure of the LAA was not appropriate for restoring SR and was not appropriate in eliminating the risk of late stroke.

Other reported complications in the literature included, but were not limited to, peripheral arterial embolism, mesenteric emboli and transient ischaemic attacks^[1]^.

Regarding specialised devices for LAA exclusion, device failure, delivery system failure, and incomplete closure of the LAA should be accounted for as potential complications, despite their higher success rates in effectively occluding the LAA^[57]^.

A further study by Romanov et al.^[58]^ compared surgical ablation using either pulmonary vein isolation (PVI) plus box lesion *versus* PVI plus box lesion plus LAA excision in patients with persistent AF; they found no clinical benefit in reducing the rate of recurrent AF by adding surgical exclusion of LAA to PVI and box lesion.

In a very large study by Yao et al.^[59]^, 75,782 patients underwent cardiac surgery. They compared surgical exclusion of LAA *versus* no surgical exclusion of LAA in patients with pre-existing AF. They concluded that concurrent surgical exclusion of LAA was associated with reduction in the risk of stroke and all-cause mortality postoperatively.

Therefore, there is room for LAA exclusion in patients with recurrent or persistent AF who remain symptomatic with heart rate control and in whom antiarrhythmic medication is no longer tolerated or is ineffective^[1]^.

#### 7. Ganglionic Plexus Ablation

Ganglionic plexus (GP) ablation achieves autonomic denervation by affecting both the parasympathetic and sympathetic components of the autonomic nervous system. GP ablation can be accomplished endocardially or epicardially^[60]^ and has been routinely adopted by minimally invasive surgical protocols as the epicardial fat pads where the GPs reside are readily accessed with ease^[61]^. The rationale for doing so is based on animal studies which have demonstrated over time that conversion of focal firing from pulmonary veins into AF is modulated by the autonomic nervous system, thus raising the possibility that destroying the GPs would influence the substrate for AF induction and perpetuation and therefore reduce incidence of arrhythmia^[62]^.

Furthermore, due to the GP being localised before ablation both visually and with high frequency stimulation, GP elimination is easily confirmed.

Despite these apparent advantages, GP ablation is increasingly being questioned. This is because autonomic ganglia can reconnect or grow over time. It has been shown that patients who have undergone GP ablation suffer higher 12-month AF relapse rates^[62]^.

#### 8. N-Contact Ablation

Contact force sensing technology allows real-time monitoring during catheter ablation for atrial fibrillation^[63]^. Despite improvements in procedural parameters, it has shown no improvement in clinical outcomes in patients with paroxysmal AF^[63]^. The experience with N-contact ablation is limited and the literature evidence is scarce, with most reported studies from single centres and of low volume. A large volume or multicentre analysis of the reported outcomes can help to understand the key outcomes behind the use of this technique.

#### 9. Hybrid Approach

The hybrid approach to the treatment of AF combines a unilateral or bilateral epicardial ablation (performed by a surgeon) with an endocardial ablation (performed by an electrophysiologist), either in a single setting or in stages^[64]^. Benefits associated with the hybrid approach include: reduced risk of tamponade during trans-septal puncture as the pericardium is left open; inadvertent injury of the phrenic nerve and oesophagus is mitigated; there is reduction in endocardial ablation, thus reducing fluoroscopy time and hence radiation and contrast load; and reduction in the occurrence of embolic events due to the lower number of endocardial ablation lines employed. The hybrid approach also allows the completion of lesion sets that cannot be performed surgically^[64]^. Table 4 outlines the advantages and disadvantages of other surgical techniques in the treatment of AF.

**Table 4:** Advantages and disadvantages of other surgical techniques in the treatment of AF.

Technique	Advantages	Disadvantages	Comments
RF ablation	Enables the formation of precise and transmural lesions by measurement of tissue resistance Does not lead to unintentional damage of surrounding structures Ease of use	Possible thrombogenesis may result from the ablation lesion lines Requires a full epicardial/endocardial box lesion	Widespread use Enables energy of frequencies of 300-1000 kHz to be generated
Cryoablation	Enables the fibrous skeleton of the heart to be maintained Reduces risk of collateral damage around the coronary arteries or AV node Associated with less risk of thrombogenesis	Time-consuming (up to 5 minutes may be needed per lesion) High recurrence rate Increased risk of damage to the oesophagus Lesions may not be successfully carried on the beating heart	Second most common method of generating required lesions that are linear, continuous and transmural in nature
Microwave	Allows deep tissue penetration, accessing full thickness not possible by radiofrequency ablation, thus enabling transmural lesions to be achieved Reduces risk of thromboembolism through avoidance of burning of the endocardial surface		Not currently used and has failed to gain popularity
Laser energy	Allows the generation of steady, delineated lesions	Not currently in use Increased risk of thrombus formation in the atria Strict angle requirement in order to target energy in a desired area	
High-energy focused ultrasound	Enables full penetration of tissue despite the surrounding fatty tissue Quick method for lesion creation Does not result in electrical gaps, leading to effective box lesions Able to overcome differing tissue thickness Reduced risk of thrombus formation		Ultrasound enables the formation of heat by oscillation of the aqueous tissue
LAA exclusion	Can be achieved either endo- and epicardially by oversewing or excision, or epicardially only by resection, ligation, stapling with or without amputation of the LAA or application of a clip system at the base of the LAA It can be performed alongside open-heart surgery but also epicardially through thoracoscopic AF ablation or as a pure stand-alone procedure, through minimally invasive means or thoracoscopically Leads to decreased rates of neurological complications Reduced risk of thromboembolism Reduced mortality rates Safe procedure	The correct technique and device must be selected in order to avoid incomplete LAA closure Failure to close LAA leads to neurological events Complete closure may not always be achieved A stump may form after the procedure and thombus formation may occur in this stump in up to 25% of patients Recanalisation may also occur. Some surgeons are reluctant to use LAA closure due to the challenge associated with managing complications that may arise from this	High failure rates are associated with the use of non-cutting stapler devices and endocardial oversewing Conversion to full sternotomy or implantation of CPB if complications arise may be needed
GP ablation	GP stimulation is thought to promote the onset of AF - ablation of the ganglionic plexus aims to target this	Major bleeding, conversion to sternotomy, cardiac tamponade and symptomatic sinus node dysfunction have been reported as complications AF recurrence has been reported post-GP ablation. Cardioversion may still be required postoperatively	Should not be performed in patients with advanced AF
Hybrid approach	Overcomes the risk of cardiac tamponade during transseptal puncture Avoids collateral phrenic nerve or oesophageal injury Negligible risk of pulmonary vein stenosis Reduced risk of cardiac emboli formation Fewer rates of arrhythmia recurrence: • Decreased rates of repeat ablation • Greater survival of AF	Lengthy intervention No difference in improvement of symptoms has been demonstrated in literature at present	This technique is still considered 'new' and no current guidelines reflect its potential use at present

AF=atrial fibrillation; AV=atrioventricular; CPB=cardiopulmonary bypass; GP=ganglionic plexus; LAA=left atrial appendage; RF=radiofrequency

In general, the safety of a hybrid surgical approach has been well demonstrated with a periprocedural mortality rate of less than 1%^[64]^. Despite its benefits, the hybrid approach is associated with some limitations. It is considered a lengthy intervention, particularly when compared to sole-surgical ablation. Also, the possibility of measuring a temporary block and bleeding of surgical dissected areas are increased and driven by the patient’s heparinisation post-transseptal puncture^[65,66]^.

Furthermore, no difference in symptom improvements has been demonstrated in the reported studies, therefore it is difficult to assess its impact on quality of life^[67]^.

Stroke and death have been reported as complications of the hybrid procedure^[67]^; alongside this, improved arrhythmia control has also been reported^[68]^. Due to this technique being more recently developed with limited evidence, current guidelines do not yet account for its risks and benefits in regard to recommendations^[1]^.

### The Future of Interventions in Atrial Fibrillation

Recent developments in both techniques and available devices have allowed AF to be targeted with a variety of multidisciplinary approaches^[66]^. Traditionally medical and surgical modalities were the norm, but with the rise of electrophysiology, a multidisciplinary approach taking into account the opinion of electrophysiologists has allowed a ‘hybrid-approach’ to develop and potentially revolutionise the way we manage AF.

The hybrid approach confers benefit by combining both percutaneous endocardial catheter ablation and minimally invasive epicardial ablation. It allows transmurality to be improved by merging endocardial and epicardial lesions^[69]^.

It is performed off-CPB solely through a thoracoscopic approach. Once finished, electrophysiologists are able to map out the systems to identify and address possible gaps, augment any non-transmural lesions and generate any additional lesions as required^[66]^.

The hybrid approach is normally carried out as a two-step process involving surgical epidural ablation first followed by catheter ablation or vice-versa. This is superior to single-catheter-based ablation (86.7% *vs*. 53.3% patients free of any atrial arrhythmia and off-antiarrhythmic drugs for hybrid and catheter-based interventions, respectively; *P*=0.04)^[70]^.

It is recommended that the epicardial procedure is performed first and then finalising with PV isolation percutaneously^[71]^. This method has been further developed so that it is also achievable as a one-step process, with success rates of 95% and 90% at 1 year for paroxysmal and persistent AF, respectively^[71]^.

Performing that which is required to eliminate AF off-CPB presents with its own challenges^[66]^. The connection to the mitral annulus through the transverse sinus is cumbersome, and there is poor visualisation behind the left atrium, increasing the risk of coronary artery damage^[66]^. There is also uncertainty regarding the use of the coronary sinus as an epicardial landmark for the mitral annulus, making the ‘Dallas lesion’ an attractive alternative, which is the line connecting to the anterior annulus at the junction of the left and non-coronary cusps of the aortic root^[66]^.

However, transmural lesions that were impenetrable by radiofrequency due to the fat-pad surrounding the dome of the left atrium and the superior vena cava can now be overcome due to the hybrid approach mapping the conduction block with an epicardial or endovascular approach^[72,73]^.

The hybrid approach has further benefits. It overcomes the risk of cardiac tamponade during transseptal puncture, as the pericardium is open. Furthermore, collateral phrenic nerve or oesophageal injury is surgically avoidable^[66]^. The risk of PV stenosis is negligible due to surgical ablation being performed on the antrum of the left atrium. It also comes with less risk of developing emboli, which are commonly linked with endocardial lesions, as epicardial induction of lesions through this method reduces this^[66]^. Other reported benefits of the hybrid approach in regard to patient outcome include: fewer rates of arrhythmia recurrence, fewer rates of repeat ablation, and greater survival^[66]^.

This is a promising and impressive approach for the future that, alongside surgical technique development and further studies, will allow us to determine its role in addressing AF.

## CONCLUSION

There has been dramatic change in the surgical management of AF, which has led to improved patient outcomes being reported. With advances in our electrophysiological understanding of AF and the recent development of the hybrid approach, encouraging outcomes are being reported at large scale which promise to revolutionise the management of AF. Moving forward with these findings, guidelines will be developed taking into account its success and limitations, enabling a standardised algorithm for targeting AF.

**Table:** 

Author's roles & responsibilities	
AH	Substantial contributions to the conception or design of the work; or the acquisition, analysis, or interpretation of data for the work; final approval of the version to be published
CB	Substantial contributions to the conception or design of the work; or the acquisition, analysis, or interpretation of data for the work; final approval of the version to be published
JSKC	Substantial contributions to the conception or design of the work; or the acquisition, analysis, or interpretation of data for the work; final approval of the version to be published
MS	Substantial contributions to the conception or design of the work; or the acquisition, analysis, or interpretation of data for the work; final approval of the version to be published
DP	Substantial contributions to the conception or design of the work; or the acquisition, analysis, or interpretation of data for the work final approval of the version to be published
ADM	Substantial contributions to the conception or design of the work; or the acquisition, analysis, or interpretation of data for the work; final approval of the version to be published
